# mGem: Submarine mycology—an analog to astromycology

**DOI:** 10.1128/mbio.01686-25

**Published:** 2025-09-23

**Authors:** Adam P. Prucka, Joan W. Bennett, Radames J. B. Cordero

**Affiliations:** 1 Department of Health Policy and Management, Johns Hopkins Bloomberg School of Public Health 25802 , Baltimore, Maryland, USA; 2 Columbia Law School, Columbia University in the City of New York, New York City, New York, USA; 3 Department of Ecology and Evolutionary Biology, Tulane University School of Science & Engineering, New Orleans, Louisiana, USA; 4 Department of Molecular Microbiology and Immunology, Johns Hopkins Bloomberg School of Public Health 25802 , Baltimore, Maryland, USA; Instituto Carlos Chagas, Curitiba, Brazil

**Keywords:** submarine mycology, astromycology, fungi in space, space microbiology, submarine microbiome, fungal contamination, closed habitat fungi, spaceflight health risks, mycotic infections in confined environments, fungal ecology spacecraft, bioaerosols in submarines, terrestrial analogs for space research

## Abstract

Submarines and spacecraft share several features that may promote the presence of fungi, including recirculated ventilation systems, moist areas, and close-quarters living. In this article, we introduce the idea of "submarine mycology" and explore how research on submarine fungi can inform the emerging field of astromycology. We highlight parallels in the fungal species present in both environments, while also noting key differences such as radiation exposure and microgravity. Arguing that submarines offer valuable lessons for spaceflight, we advocate for renewed research using modern genetic tools to characterize submarine fungi.

## PERSPECTIVE

Astromycology, the study of fungi in space environments, has emerged as a critical field for understanding both their risks and potential applications (1). Among the most pressing concerns is the protection of astronaut health against mycotic diseases, which are often difficult to diagnose, challenging to treat, and lack effective prophylaxis (2). To expand this field and better understand the health risks posed by fungi in space habitats, we can look to research in other enclosed environments. Nuclear submarines are a compelling analog because their recirculated atmospheres and extended human occupancy bear a striking resemblance to spacecraft (3), while being far more accessible. Furthermore, their fungal ecology has been studied for decades, constituting a substantial body of research that we refer to as “submarine mycology” (4–8). We postulate that submarine mycology can enhance our understanding of fungal behavior in human-occupied, enclosed systems, informing the burgeoning field of astromycology and supporting astronauts’ health, particularly on long-duration missions.

**Fig 1:**
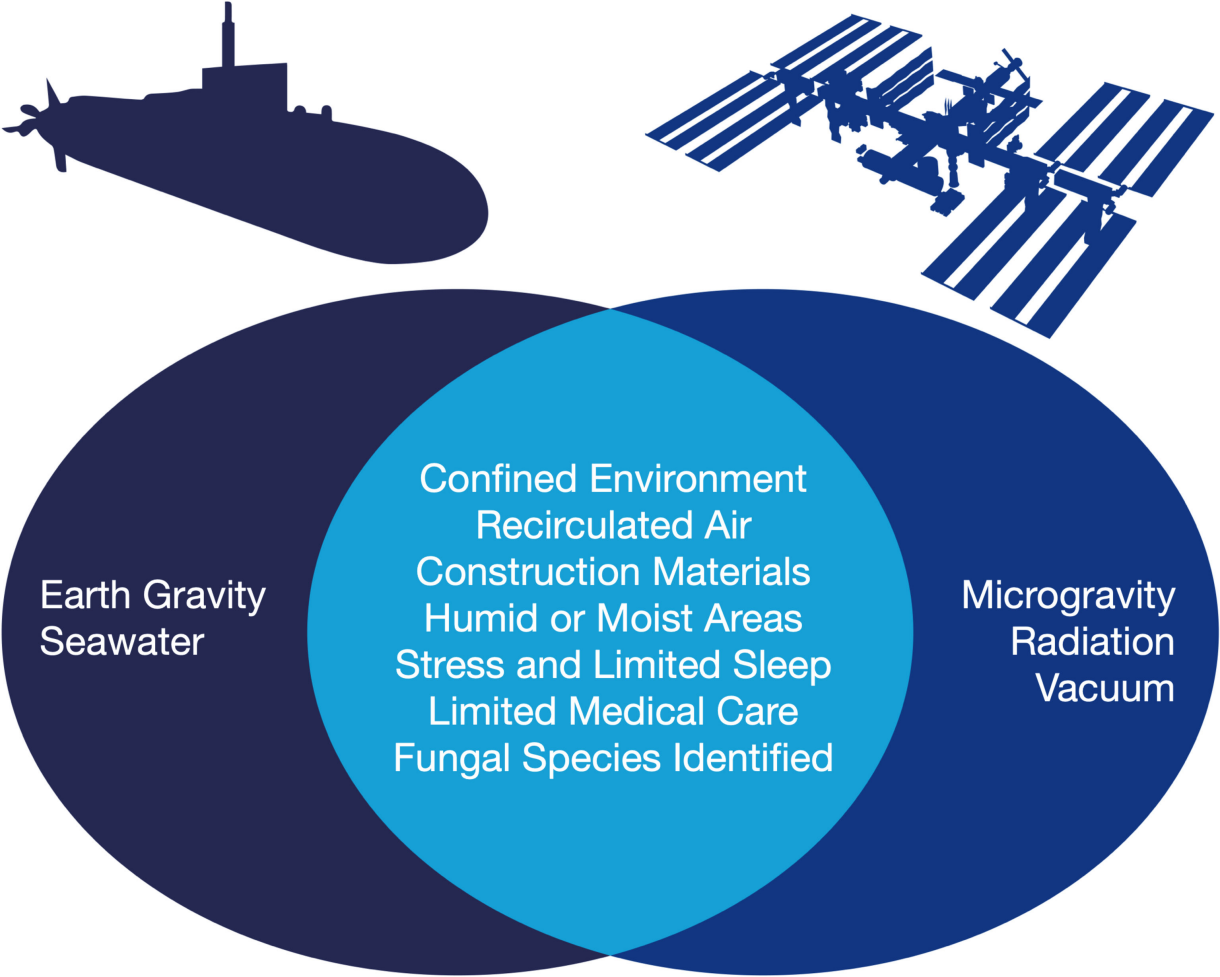
Environmental and operational parallels and differences between submarines and spacecraft relevant to fungal persistence. Submarines operate under Earth’s gravity and are surrounded by seawater, while spacecraft operate under microgravity, are exposed to cosmic radiation, and are surrounded by the vacuum of space. Nevertheless, both vehicles share conditions that promote fungal persistence, including crew confinement, recirculated air, the utilization of materials prone to degradation, humid or moist areas, stress and limited sleep, and limited medical care. The identification of overlapping fungal taxa—including members of *Aspergillus*, *Penicillium*, and *Fusarium*—in both vessels underscores the relevance of submarines as an analog environment for studying fungal risks in spaceflight.

Submarines are a strong analog to spacecraft, as exemplified by the comparative graphic presented in Fig. 1. Each vessel consists of a confined tube that must sustain human survival for extended periods of time within a hostile external environment, whether the high-pressure depths of the ocean or the vacuum of space. Although high-efficiency air filtration systems are used in both vehicles, fungal spores can still persist in their recirculated atmospheres, creating challenges in microbial control during long-duration missions (3, 5, 6). The polymers, foams, and textiles that comprise these vessels can serve as substrates for fungal colonization and degradation (9–11), and moisture is present to facilitate fungal growth, whether from air conditioning condensate in submarines (7) or from wet towels in spacecraft (10). Compounding these concerns, sleep disruptions can heighten the crewmembers' susceptibility to infectious diseases (12–16), and the voyagers must rely on autonomous medical care to fight any resultant illnesses (17, 18). There are, however, clear differences in the environmental contexts of these vessels, for spacecraft experience microgravity and cosmic radiation, whereas submarines operate under Earth’s gravity and are surrounded by consistently damp conditions. Nevertheless, despite these distinctions, both manned vehicles are well-suited to serve as fungal habitats, underscoring the potential for submarine mycology to be used as a model to advance our understanding of fungal risks during spaceflight.

The composition of fungal species aboard military submarines exhibits two principal lessons for the space environment. First, the presence of opportunistic fungi in undersea vessels, including *Aspergillus* and *Fusarium* spp*.*, substantiates the previous identification of these taxa aboard the International Space Station (19) and confirms the potential for these organisms to persist in spacecraft and pose health hazards to astronauts (6, 7). Therefore, aerospace physicians should be prepared to treat fungal diseases, especially superficial infections, with the 2003 study of Vakulova et al. study reporting mycoses among 41.2% of Russian submariners, principally *Candida albicans* and nail and foot dermatophytes (20). However, these rates of fungal infections can vary significantly among vessels, with Hinshaw’s 1967 study reporting only 2.95 superficial mycoses, 0.22 intermediate mycoses, and 0.22 deep mycoses per 1,000 U.S. submariners (5). The vast majority of Hinshaw’s mycoses (87.2%) were superficial with a small incidence of intermediate and deep mycotic infections (5). These findings from submarines reinforce the importance of treating fungal diseases in space, building on previous studies that demonstrate the presence of opportunistic microbes in spacecraft and the susceptibility of astronauts to microbial infections (21). Indeed, the health risks may be even greater in space, since the space environment can promote fungal virulence, biofilm formation, and antifungal resistance (19, 22–24) and can also suppress astronauts’ immune systems, raising the likelihood of infection (25, 26). Underscoring these risks, astronauts have already reported both *C. albicans* infections (27) and dermatophytoses (28). In addition, mycotoxigenic fungi have been isolated from spacecraft, many of which have been isolated aboard submarines as well, including *Aspergillus*, *Penicillium*, and *Fusarium* species (4, 6, 7, 29).

Second, since the fungal species observed in submarines are not constant between vessels or across time, space agencies should be prepared to tackle a wide diversity of unpredictable fungal threats. This variation is illustrated in Table 1, which lists the fungal taxa identified by Milroy and Duffner (4), Doris et al. (6), and Upsher et al. (7) on U.S., British, and Australian military submarines, respectively. The final column of Table 1 uses Nickerson’s 2024 review paper to denote whether these taxa have been identified on the International Space Station, and this comparison showcases significant overlap between submarine mycology and astromycology (19). While Upsher et al. found that *Aspergillus* predominated in Australian submarine atmospheres (7), Doris et al. reported that *Penicillium* comprised 96% of the fungi in British submarine atmospheres (6), and Milroy and Duffner observed that *Penicillium* made up nearly 50% of the fungal colonies identified on settling plates from American submarines (4). Additionally, the fungal diversity in submarines fluctuates over time—in Kelley’s 1967 study, the diversity of fungi at the beginning of the voyage eventually transitioned into a near *Penicillium* monoculture (8). These findings suggest that enclosed vessels foster distinct and dynamic microbial ecologies shaped by localized environmental conditions, underscoring the need for continuous fungal surveillance and robust antifungal strategies. Indeed, since a wide range of fungal species have already been observed on the International Space Station (1, 10, 19, 30–37), spaceflight contingency planners should be prepared to tackle a broad spectrum of fungal threats as missions extend farther and longer into space.

**TABLE 1:** Fungal taxa identified in military submarines and on the International Space Station^*a*^

Fungus	Milroy and Duffner (4)	Doris et al. (6)	Upsher et al. (7)	Observed on International Space Station (Table S1 in Nickerson et al. [19])
*Aspergillus* spp.	x	x	x	x
*Aspergillus fumigatus*			x	x
*Aspergillus flavus*			x	x
*Aspergillus glaucus*			x	
*Aspergillus niger*		x	x	x
*Aspergillus ochraceus*			x	
*Aspergillus versicolor*		x		x
*Cephalosporium/Acremonium*		x (as *Cephalosporium*)		x (as *Acremonium*)
*Cladosporium* spp.		x	x	x
*Fusarium*		x	x	x
*Geotrichum*			x	
*Mucor*		x	x	
Osmophilic fungi			x (identity not specified)	x (especially *Wallemia semi*, *Aspergillus penicillioides*, and *Eurotium*)
*Paecilomyces*			x	
*Penicillium*	x	x	x	x
*Phoma*		x	x	x
*Rhizopus*	x	x	x	
*Rhodotorula*			x	x
Yeast (identity not specified)	x	x	x	x

^*a*^ Species names reflect their reported identifications in the cited studies. However, many of these identifications were based on morphological traits alone and likely refer to members of taxonomic sections such as *Aspergillus* sections Fumigati, Flavi, and Nigri, rather than confirmed species-level assignments. An “x” indicates that the fungal taxon in the row was reported in the reference source denoted by the column.

Submarine mycology underscores the importance of adequate ventilation systems in mitigating airborne fungal threats. While visible fungi are present within submarines, the levels of aerial fungal spores are usually quite low and tend to decline over the course of the voyage (4, 6). This outcome is largely attributed to the ventilation system, which operates through two mechanisms: the use of high-efficiency air filters and “wall loss”, wherein spores deposit within the ventilation ducts rather than escaping through the wall outlets (4, 6, 7). However, data from submarine mycology also demonstrate that ventilation alone cannot abrogate all fungal risks. Surface cleaning and other on-board physical activities can disturb accumulated dust and temporarily overwhelm the ventilation system, yielding a marked increase in airborne fungal spores (4, 7). Dermatophytic fungi likewise persist (4, 5), as exemplified by athlete’s foot (tinea pedis) (4, 38, 39), the single most common skin mycosis reported by Milroy and Duffner (4). Furthermore, despite the low levels of aerial fungal spores, submariners have experienced symptoms of allergies and sick building syndrome (SBS), including elevated eosinophil counts and headaches (4, 6–8). Recent studies show that fungi not only produce mycotoxins (29) but also emit biologically active volatile organic compounds (VOCs) into the air, which may contribute to SBS (40–44). Hence, even if ventilation removes allergenic spores, fungi may still have negative effects on human health in both submarines and spacecraft through their emission of VOCs and other airborne metabolites.

Submarine mycology has also revealed the spatial distribution of fungi within underwater vessels, providing valuable insights into the highest-risk zones within spacecraft and helping to identify key areas for targeted fungal control strategies. For example, fungi thrive in locations rich in carbon sources and organic matter, such as charcoal air filters, food preparation areas, and bedsheets containing exfoliated skin (4, 6, 7). Damp environments, including drains, showers, air conditioning units, and pipe condensate are also common habitats for fungi, especially yeasts (4, 6, 7). Certain filamentous genera, including *Cladosporium*, *Fusarium*, and *Cephalosporium* have also been observed colonizing and degrading the interior surfaces of submarines (6). To mitigate this issue, the U.S. Navy has developed specialized coatings to inhibit mold and mildew (11). A similar approach may be advisable for spacecraft as well, particularly since black fungi have already been observed growing on the surface of a damp panel in the International Space Station’s gym (9, 10, 19).

Submarine mycology has limitations as an analog to astromycology. The high levels of radiation in space (45) may induce greater levels of mutation in fungi (46–50), and submarines’ aquatic surroundings may favor the presence of mold far more strongly than the vacuum of space. Furthermore, because astronauts do not walk in microgravity, foot traffic will not disturb fungal spores (4). Despite these caveats, submarine mycology remains a compelling model for astromycology, providing insights into the microbiota of sealed environments. Since most of the relevant studies are decades old and may not reflect current conditions aboard submarines, we urge researchers to conduct modern phylogenetic, metagenomic, and DNA-based analyses similar to those conducted on the International Space Station (32), enabling updated parallels to be drawn between submarines and spacecraft. Such research may also be more cost-effective than cognate studies done in microgravity aboard spacecraft. In this way, submarines can provide a timely and practical platform for anticipating fungal risks on long-duration spaceflight, enhancing astronaut safety and promoting the health of spacefarers during extraterrestrial missions.
